# MET Activation in Lung Cancer and Response to Targeted Therapies

**DOI:** 10.3390/cancers17020281

**Published:** 2025-01-16

**Authors:** Sarah Anna Okun, Daniel Lu, Katherine Sew, Asha Subramaniam, William W. Lockwood

**Affiliations:** 1 Integrative Oncology, BC Cancer Research Institute, Vancouver, BC V5Z 1L3, Canada; sokun@bccrc.ca (S.A.O.); ksew@bccrc.ca (K.S.); asubramaniam@bccrc.ca (A.S.); 2 Interdisciplinary Oncology Program, University of British Columbia, Vancouver, BC V6T 1Z4, Canada; 3 Department of Pathology, Memorial Sloan Kettering Cancer Center, New York, NY 10065, USA; luc4@mskcc.org; 4 Department of Pathology and Laboratory Science, University of British Columbia, Vancouver, BC V6T 1Z4, Canada

**Keywords:** MET, oncogene, lung cancer, targeted therapy, resistance mechanisms

## Abstract

The hepatocyte growth factor receptor (MET) is a receptor tyrosine kinase (RTK) that mediates the activity of a variety of downstream pathways upon its activation. These pathways regulate various physiological processes within the cell, including growth, survival, proliferation, and motility. Under normal physiological conditions, this allows MET to regulate various development and regenerative processes; however, mutations resulting in aberrant MET activity and the consequent dysregulation of downstream signaling can contribute to cellular pathophysiology. Mutations within MET have been identified in a variety of cancers and have been shown to mediate tumorigenesis by increasing RTK activity and downstream signaling. In lung cancer specifically, a number of patients have been identified as possessing MET alterations, commonly receptor amplification (METamp) or splice site mutations resulting in loss of exon 14 (METex14). Due to MET’s role in mediating oncogenesis, it has become an attractive clinical target and has led to the development of various targeted therapies, including MET tyrosine kinase inhibitors (TKIs). Unfortunately, these TKIs have demonstrated limited clinical efficacy, as patients often present with either primary or acquired resistance to these therapies. Mechanisms of resistance vary but often occur through off-target or bypass mechanisms that render downstream signaling pathways insensitive to MET inhibition. This review provides an overview of the therapeutic landscape for MET-positive cancers and explores the various mechanisms that contribute to therapeutic resistance in these cases.

## Simple Summary

The hepatocyte growth factor receptor (MET) facilitates cell growth, survival, proliferation, and motility. Alterations in MET result in the dysregulation of its activity and contribute to cancer development and progression. Various therapies have been developed to treat MET-positive cancers; however, therapeutic response rates are relatively poor compared to other kinase inhibitors due to patients displaying primary and acquired resistance. This review explores MET dysregulation in cancer, the available treatment options for MET-driven cancers, and the mechanisms that lead to resistance to these therapeutics.

## 1. Introduction

Lung cancer is the leading cause of cancer mortality worldwide, responsible for almost a fifth of all cancer-related deaths annually [1,2]. Lung cancer is histologically classified as either small cell or non-small cell lung cancer (SCLC and NSCLC, respectively), with the latter being further categorized as lung adenocarcinoma (LUAD), squamous cell carcinoma (SqCC), or large cell carcinoma (LCC). In a subset of NSCLC patients, alterations affecting the hepatocyte growth factor receptor (MET) have been identified, which have been shown to contribute to the development and progression of disease.

**Figure 1:**
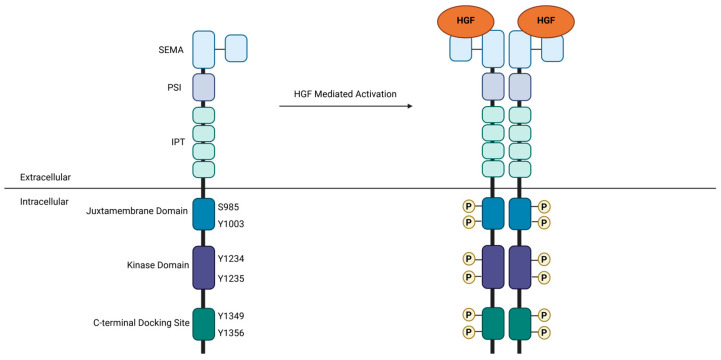
Structural organization of MET. The extracellular region is made up of the semaphorin (SEMA) domain, the plexin-semaphorin-integrin (PSI) domain, and four immunoglobulin-plexin-transcription (IPT) domains, which facilitate ligand binding and receptor dimerization. The intracellular region contains the juxtamembrane domain, the kinase domain, and the C-terminal docking site; these regions are involved in the regulation of MET activity and the activation of downstream signaling cascades. Receptor activation occurs upon the binding of hepatocyte growth factor (HGF), resulting in receptor dimerization and a series of transphosphorylation events.

MET is a receptor tyrosine kinase (RTK), which facilitates the activation of a variety of molecular pathways and consequently mediates numerous physiological and pathophysiological processes. Under normal physiological conditions, receptor activation is dependent upon binding to its cognate ligand, hepatocyte growth factor (HGF), a heterodimeric protein that binds MET extracellularly. The extracellular region of MET contains three domains—the semaphorin (SEMA), plexin-semaphorin-integrin (PSI), and four immunoglobulin-plexin-transcription (IPT) domains, which facilitate ligand binding, and receptor maturation and dimerization [3,4,5]. Ligand binding induces dimerization and consequent transphosphorylation events within the intracellular region, which allow for the regulation of MET activity through interaction with negative regulators and downstream effectors. The intracellular region of MET is likewise composed of three domains: (1) the juxtamembrane domain, which contains residues S985 and Y1003 involved in the negative regulation of MET activity following ligand activation [6,7]; (2) the kinase domain, which is transphosphorylated at residues Y1234 and Y1235 and adopts an active conformation following ligand-mediated receptor dimerization [8]; and (3) the C-terminal multi-substrate docking domain, which is phosphorylated at residues Y1349 and Y1356 to create binding sites that recruit downstream secondary messengers and adaptor proteins [9] (Figure 1). Key interactors include growth factor receptor-bound protein 2 (Grb2), which in turn recruits the guanine exchange factor SOS1 necessary to activate RAS-MAPK signaling, as well as Grb2 associated binding protein 1 (Gab1), which acts as an adapter protein for downstream effectors to indirectly interact with MET [10,11]. Secondary messengers such as phosphoinositide 3-kinase (PI3K) and the signal transducer and activator of transcription 3 (STAT3) can also directly associate with the phosphorylated MET receptor through their SH2 domain [12,13]. The sum of these interactions results in the initiation of downstream signaling cascades, which ultimately promote cell survival, proliferation, and motility. While these processes establish MET as a crucial mediator of development and homeostasis under normal physiological conditions, their dysregulation can easily transform MET into a potent driver of tissue pathophysiology and disease.

**Figure 2:**
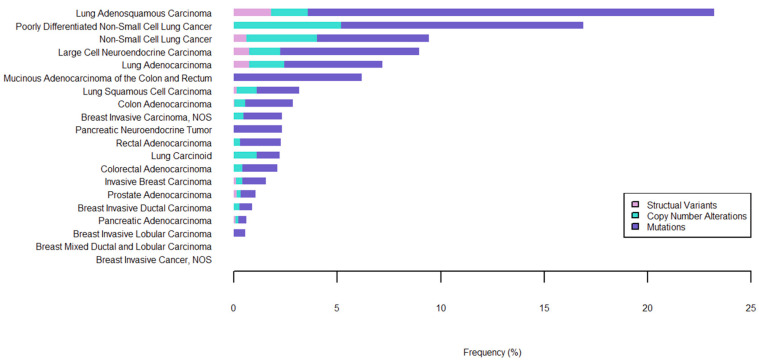
Frequency of MET alterations in solid cancer types. Alterations include mutations, copy number alterations, and structural variants (including fusions). Data from MSK-CHORD (MSK, Nature 2024) [25].

MET alterations that contribute to dysregulation of its activity—namely its overactivation—have been identified in a variety of cancers, and these alterations contribute to the development and progression of disease (Figure 2). In NSCLC, the amplification of the MET gene (METamp) has been observed in 1–3% of cases and has been associated with poorer patient prognosis and resistance to targeted therapies [14,15,16,17,18]. MET copy number alterations can arise from focal amplification of the gene itself or through polysomy of chromosome 7, both of which result in increased MET expression and signaling activity. Additionally, aberrant activation can be caused by exon skipping mutations resulting in the deletion of exon 14 (METex14); the resultant RTK exhibits impaired downregulation following ligand activation and increased receptor half-life. METex14 is observed in about 3% of NSCLC cases [19,20,21], and while deletions of this exon can be the result of genomic deletion events, they are more commonly caused by mutations in splicing donor and acceptor sites or within the polypyrimidine tract [21,22,23]. Exon 14 encodes for the majority of the juxtamembrane domain and encompasses the Y1003 residue, which is necessary for the regulation of MET activity. Upon receptor activation, Y1003 is phosphorylated, allowing for its recognition by E3 ubiquitin ligase Casitas B-lineage lymphoma (CBL) and the subsequent ubiquitination of MET; ubiquitination in turn signals for MET’s degradation, and ultimately maintains MET at appropriate levels within the cell [7,24]. When MET levels become abnormally elevated—whether through amplification or exon 14 deletion—the resultant increase in MET signaling results in the hyperactivation of pathways that lead to increased proliferative and metastatic potential.

Attempts to combat the effects of METamp and METex14 in cancer progression have led to various therapies being developed, which include small molecule tyrosine kinase inhibitors (TKIs) (Table 1). While TKIs can be differentially classified based on their specific mechanisms of action, they all exert their effects by binding the receptor and preventing its activation. Type I MET TKIs act as competitive inhibitors by binding the receptor in its active conformation at the ATP binding pocket; therapies that fall under this classification include crizotinib, a type IA TKI, and capmatinib and tepotinib, which are type IB TKIs. While both classes bind the ATP-binding pocket, they vary in that crizotinib additionally interacts with the G1163 residue and displays activity against other RTKs, such as ALK and ROS1 [26,27]. Type II TKIs, such as cabozantinib and merestinib, similarly inhibit ATP binding but do so by interacting with the inactive conformation of the receptor. The final class of MET inhibitors is type III TKIs, which prevent receptor activation by binding to an allosteric site outside of the ATP binding pocket. Thus far, tivantinib is the only type III MET TKI that has been developed, but it has yet to gain clinical approval for the treatment of METamp- or METex14-positive NSCLC. While tivantinib has displayed potent activity against MET, it has furthermore demonstrated cytotoxicity in MET-independent cell lines, suggesting it is not entirely MET-specific; outside of MET, tivantinib has demonstrated activity against NLRP3, and it has been shown to disrupt microtubule dynamics [28,29,30].

Besides TKIs, antibodies have also been developed to therapeutically target MET in cancer. This includes onartuzumab, a monoclonal antibody that inhibits MET binding to HGF, and emibetuzumab, a bivalent antibody capable of inhibiting both HGF-dependent and -independent MET activation by preventing ligand binding and promoting receptor internalization and degradation [46,47]. Unfortunately, neither has demonstrated significant benefit in clinical trials [48,49]. Antibody–drug conjugates (ADCs) have also been developed, such as telisotuzumab-vedotin, which is a combination MET antibody and anti-microtubule drug that has shown potential benefit in clinical trials for patients with MET-positive cancers [50,51]. Furthermore, there is research being conducted to target MET using chimeric antigen receptor T cells (CAR-T cells); however, while this approach shows promise, further investigation is required before it can be clinically evaluated [52,53].

At the time of this review, multiple TKIs have received approval for the treatment of NSCLC patients harboring METex14 or METamp. Crizotinib received a breakthrough therapy designation from the FDA following the results of the PROFILE 1001 trial, which assessed this TKI in METex14-positive patients; it achieved an overall response rate (ORR) of 32% (95% CI, 21 to 45) and a progression-free survival (PFS) of 7.3 months [32]. This was followed by approval for capmatinib and tepotinib, based on results from the GEOMETRY mono-1 and VISION trials, respectively. Capmatinib achieved an ORR of 41% (95% CI, 29 to 53) in METex14-positive patients who had previously received other lines of treatment, while in treatment-naïve patients, the ORR was found to be 68% (95% CI, 48 to 84) [33]. For patients with METamp, the ORR for capmatinib was 29% (95% CI, 19 to 41) in previously treated patients and 40% (95% CI, 16 to 68) in the treatment-naïve group [33]. In the VISION trial, tepotinib achieved an ORR of 46% (95% CI, 36 to 57) in METex14-positive patients and a median duration of response of 11.1 months [38]. For the aforementioned therapies, edema and nausea were the most common treatment-related adverse effects (TRAEs), with the majority of TRAEs being grade 1 or 2. Savolitinib has additionally received approval for the treatment of METex14-positive NSCLC patients in China, based on results from a phase II trial, where it achieved an ORR of 49.2% (95% CI, 36.1 to 62.3) after a median follow-up time of 17.6 months; all patients reported TRAEs, with 46% of patients displaying TRAEs of grade 3 or more [36].

While able to reduce MET signaling activity, patients who are prescribed these therapies often develop resistance; methods of therapeutic resistance vary but ultimately limit the efficacy of currently available targeted therapies. A better understanding of MET signaling and the mechanisms underlying resistance to targeted MET therapies is, therefore, imperative for the design of more robust treatment plans and improving patient prognosis.

## 2. MET Signaling

### 2.1. Normal Physiological Function

MET’s involvement in proliferation and motility signaling makes it an indispensable mediator of embryonic development and tissue regeneration. Its importance in the former has been demonstrated using mouse models, where the loss of Hgf-Met signaling impairs placental development and trophoblast differentiation and ultimately results in lethal embryonic abnormalities [54,55]. For example, the loss of Met signaling impacts the development of the early limb bud and diaphragm, interrupting the migration of myogenic precursors and preventing skeletal muscle development in these areas [56]. Furthermore, the expression of both HGF and MET can be observed at different time points and developing tissues in mouse, rat, and human embryos, forming specific spatiotemporal expression patterns crucial for organogenesis [57,58,59]. This signaling axis has demonstrated particular importance in the growth and development of the liver, as mouse embryos homozygous for non-functional Hgf or Met display a marked reduction in liver development and size relative to wild-type littermates, further contributing to the embryonic lethal phenotype [55,56]. This is due to MET’s role in tubulogenesis, a physiological process necessary for the formation of various organs during embryonic development. This role for MET was initially suggested following the observation that the addition of HGF is able to induce tubulogenic activity in Madin–Darby canine kidney (MDCK) cells grown in culture [60]. Further in vitro studies have confirmed a role for MET in renal tubulogenesis in particular, supporting roles for this receptor in renal development and regeneration following injury [61,62,63]. In vivo studies have additionally described roles for MET in these processes; for example, the intravenous injection of HGF in mice demonstrated positive effects on tubulogenesis and regeneration in response to kidney failure [64]. A role for Met in tubulogenesis has also been described in other organ systems, such as the mammary glands, where the addition of HGF has been shown to stimulate branching morphogenesis [65,66,67].

Post-development, MET plays a key role in guiding tissue regeneration and wound repair. Its involvement following tissue injury has been demonstrated in various organs. For example, treatment with MET agonist HGF prevents renal dysfunction following kidney injury by promoting renal tubular cell proliferation [61,64]. Following partial hepatectomy, conditional Met knockout in mice impaired liver regeneration and was associated with the impaired cell cycle progression and motogenic capacity of hepatocytes [68,69]. Similarly, conditional Met knockout in epidermal cells resulted in significantly delayed wound healing in vivo [70]. In vitro, scratch assays following up on these results suggested that Met facilitates this process in part by helping to orient keratinocytes toward the wound [70]. Finally, Met signaling has been implicated in peripheral nervous system regeneration, in particular through its ability to mediate mitogen-activated protein kinase (MAPK) signaling [71]. Following injury, it was observed that Hgf expression is elevated at the site of injury and that the therapeutic inhibition of Met results in decreased myelination and overall regenerative capacity [71]. Inhibition of Met signaling following nerve injury was further shown to impede axon outgrowth and decrease levels of c-Jun, a transcription factor involved in axonal regeneration [72,73].

### 2.2. Oncogenic Signaling

MET facilitates complex developmental and homeostatic processes through its ability to integrate growth, survival, and migration cues from the extracellular environment. This necessitates the use of diverse signaling pathways, tightly regulated via dedicated signal transducers, adaptors, and scaffolding proteins to collectively modulate receptor activity and signal transduction. When aberrantly activated, the same pathways can promote invasive growth and metastatic behavior in cancer cells. MET’s role in cancer was initially identified in a chemical mutagenesis screen of a human osteosarcoma cell line, which yielded a fusion protein with transforming potential [74]. Further investigation revealed that the fusion protein contained a translocated promoter region (Tpr), and the kinase and docking domain of MET; the Tpr contains a leucine zipper domain that leads to the dimerization of the fused protein and the constitutive activation of the MET kinase domain [75,76]. Involvement of the MET receptor or its ligand HGF has been subsequently described in various solid malignancies, where its signaling contributes to tumor progression and is associated with poor patient survival. Studies have also reported extensive crosstalk between the HGF–MET axis and other signaling pathways such as VEGF, EGFR, and IGF-1R, as well as developmental pathways like Wnt/β-Catenin. This cooperation has been shown to enhance tumor progression and/or resistance by promoting angiogenesis and an epithelial-mesenchymal transition (EMT) towards an invasive phenotype [77,78,79]. For example, the MET promoter can be activated under hypoxic conditions by HIF-1α, providing tumors a way to escape anti-angiogenic therapy using VEGF inhibitors. These tumors often acquire a more invasive phenotype and require dual VEGF and MET inhibition to synergistically induce prolonged anti-tumor response [80,81,82,83].

**Figure 3:**
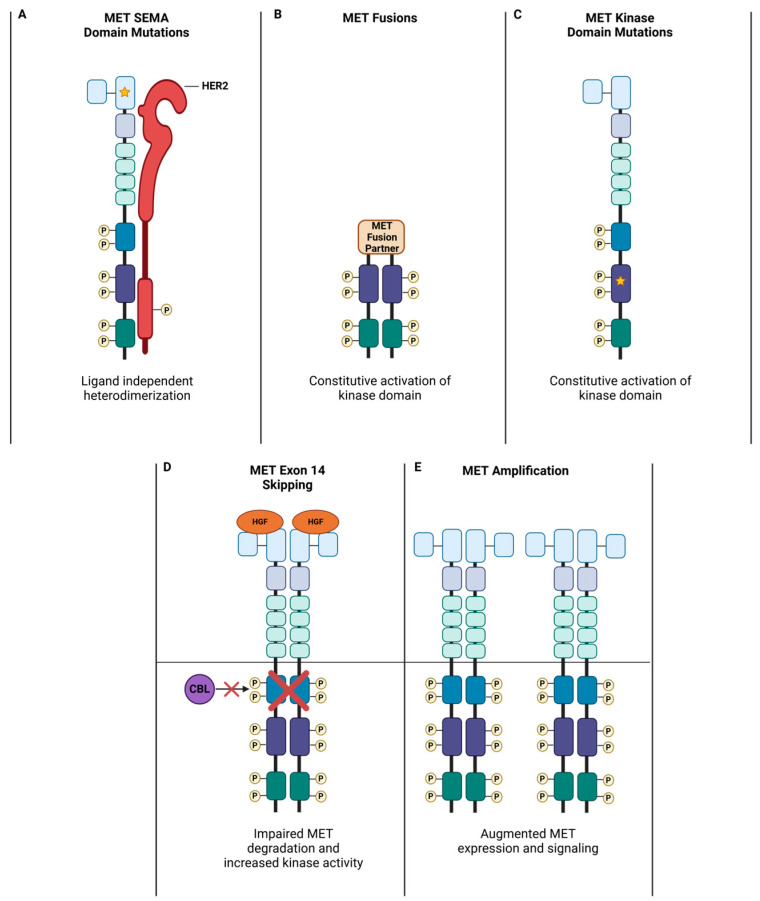
MET alterations observed in cancer. (**A**) SEMA domain mutations (e.g., N375S) decrease MET’s ligand affinity and increase HER2 heterodimerization. (**B**) MET fusions result in the loss of ligand binding and regulatory domains, resulting in increased signaling activity. (**C**) Kinase domain mutations (e.g., M1268T and Y1248H) lead to constitutive kinase activity. (**D**) Skipping of exon 14 impairs MET degradation, leading to increased half-life and signaling activity. (**E**) Amplification results in elevated MET levels and activity within the cell.

Alterations that give rise to MET’s transforming potential vary extensively and appear to correlate with cancer type (Figure 3). As mentioned earlier, gene amplification or copy number gain is a commonly reported genomic event leading to MET overexpression and ligand-independent activation, which has been shown to confer transformative potential in various cancers including glioblastoma [84], NSCLC [85], colorectal cancer [86], and medulloblastoma [87]. HGF upregulation by cytokines such as TNF-α, IL-1, EGF, and fibroblast growth factor can generate an autocrine receptor activation loop to similarly hyperactivate MET signaling [88,89]. However, MET-activating mutations have garnered the most intense clinical interest, as they serve as strong predictive biomarkers to anti-MET targeted therapies. In hereditary papillary renal carcinoma (HPRC), where oncogenic MET mutations were first described, missense mutations clustered in the kinase domain region led to increased kinase activity [90]. Interestingly, most of the reported kinase domain mutations (e.g., M1149T, V1206L, L1213V, V1238I) display weak oncogenic potential, requiring HGF stimulation to confer sufficient MET phosphorylation to drive transformation [91]. This may explain why patients carrying these mutations in the germline tend to develop HPRC only later in life [90]. In contrast, two strongly activating variants (M1268T, Y1248H) with constitutive kinase activity exhibited HGF-independent behavior [91], pointing to a need to assess HGF dependency on a per-variant basis. Increasingly, MET alterations outside the kinase domain have been described in non-HPRC cases. For example, germline mutations in the SEMA domain (e.g., N375S) have been observed in NSCLC patients and have been shown to alter ligand affinity for the receptor [92,93,94]. Paradoxically, the N375S mutant exhibits decreased binding to HGF, instead conferring enhanced ligand-independent binding affinity to HER2 [95]; cancers with this mutation are insensitive to MET TKI treatment but may respond well to HER2-targeted therapy. These findings demonstrate the truly polymorphic nature of the MET receptor, underscoring a need to characterize the complex relationship between MET structure, function, and disease biology.

Somatic events involving MET have also been described in NSCLC and other cancers, with the most common being exon 14 deletion mutations that result in loss of the juxtamembrane regulatory domain. This region encompasses multiple regulatory sites, most prominently the Y1003 phosphorylation residue that binds c-CBL to promote receptor ubiquitination and downregulation [96]. While ubiquitination does not impact receptor internalization, it helps regulate the balance between receptor recycling and degradation by trafficking the internalized MET toward the lysosomes [24]. MET is known to continue signaling while localized within its endosomal compartments following endocytosis, with evidence suggesting this compartmentalization of internalized receptors plays an important role in spatially modulating signal transduction processes within the cell [97,98]. For instance, MET endocytosis is required for Rac1-driven motility [99]. As well, the loss of the recycling adaptor GGA3 has been shown to enhance MET trafficking into degradative compartments, leading to the attenuation of ERK1/2, but not AKT, phosphorylation following HGF stimulation [24,100]. Since altered MET recycling dynamics have been observed for other activating mutations [99], the absence of Y1003 phosphorylation, given its direct involvement in endocytic trafficking, may similarly impact MET spatiotemporal signaling and partner recruitment. Interestingly, studies have shown that mutating this residue is sufficient to confer oncogenic behavior that phenocopies the loss of the entire exon [96,101,102]. However, Y1003 point mutations are rarely detected in the clinic, with the majority clustered at splice sites upstream or downstream of exon 14. This points to a potential tumor-suppressive role for other regulatory sites located in this domain, with S985 and D1000 being the most extensively studied. The S985 residue is phosphorylated by protein kinase C (PKC-δ and -ε) in response to oxidative stress and dephosphorylated by PP2A [6,103]. S985 phosphorylation dampens responsiveness to HGF stimulation, potentially abrogating MET’s anti-apoptotic function in response to elevated ROS levels, leading to cell death [103,104]. Loss of S985 phosphorylation could represent a hypothetical mechanism for tolerating ROS thresholds that normally trigger senescence, apoptosis, or ferroptosis, helping to pave the way toward oncogenic transformation [105,106]. In contrast to the Y1003 and S985 phosphorylation sites, the D1000 aspartic residue serves as a Caspase 3 cleavage site in the juxtamembrane region [107]. MET can be proteolytically cleaved at specific sites when cells experience apoptotic stress, separating the extracellular ligand-binding domain and the intracellular kinase domain [108,109]. The resultant intracellular fragment (p40) localizes to the ER and mitochondrial membrane to stimulate Ca^2+^ release by the ER and uptake by the mitochondria [109]. This is mediated through its association with pro-apoptotic protein BAK and leads to the permeabilization of the mitochondrial membrane and the release of Cytochrome C. The absence of the D1000 cleavage site in METex14 mutants thus abrogates one of the few known pro-apoptotic roles of MET signaling [108].

### 2.3. MET Alterations as a Primary Driver of NSCLC

Both METamp and METex14 occur independently in NSCLC, and they have been found to co-exist in some cases; in such instances, it is possible that co-occurrence could impact tumorigenesis and response to targeted therapies; however, larger study cohorts and further investigation are needed to confirm any possible correlation [20,21,110,111].

**Figure 4:**
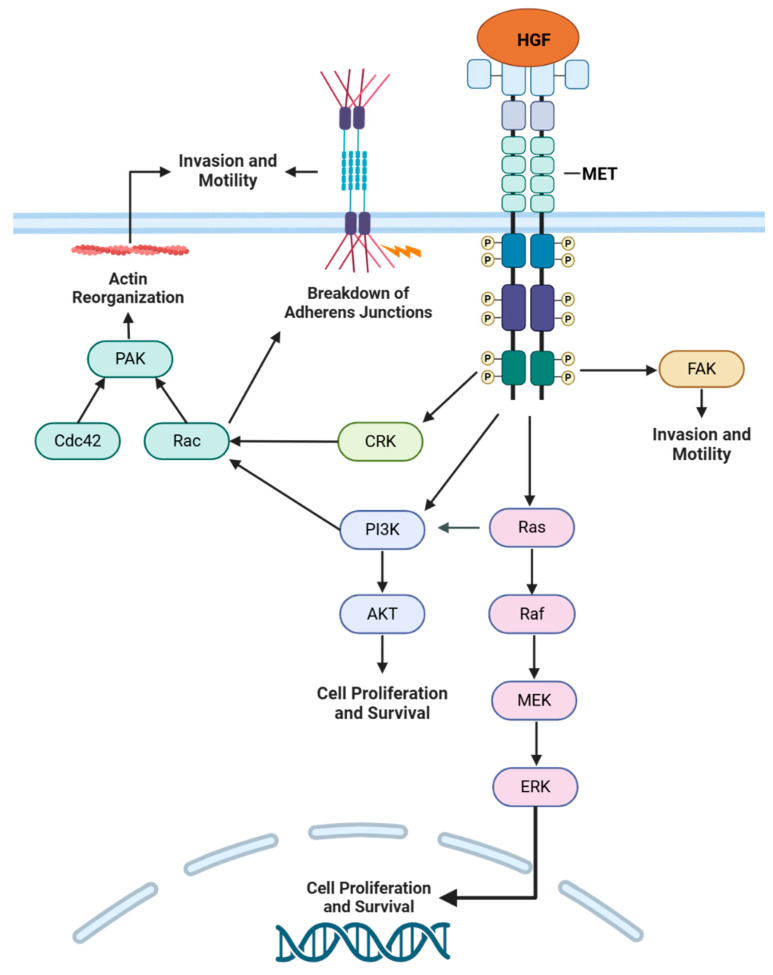
MET signaling facilitates various processes that contribute to pathogenesis. Activated MET leads to the activation of the PI3K and MAPK pathways, which promote cell proliferation and survival. MET is also able to activate FAK and CRK, resulting in greater cell motility and invasion.

Due to its ability to mediate the activation of proliferative signaling pathways, aberrant MET activation can promote the growth and survival of cancer cells, leading to oncogene dependence (Figure 4). In cancer cell lines displaying MET overexpression, the shRNA-targeted inhibition of MET signaling significantly impaired cell viability [112,113]; in NSCLC cell lines specifically, the inhibition of MET in METamp lines led to growth inhibition characterized by a block in the cell cycle at the G1-S transition [114]. Additional mechanisms of increased cell survival could be attributed to the anti-apoptotic effects of MET signaling, namely activation of the PI3K pathway and AKT [115].

MET’s role as a mediator of cell motility can be hijacked in an oncogenic context to further promote tumorigenesis by facilitating tumor invasion and metastasis. MET signaling has demonstrated an ability to mediate these processes in various facets; for instance, the addition of HGF promotes adhesion to extracellular matrix components and the activation of focal adhesion kinase (FAK) that, when inhibited, downregulates HGF-driven cell migration [116,117]. Through the recruitment of CRK adaptor proteins and the upstream activation of PI3K and MAPK signaling, MET can disrupt the formation of adherens junctions, initiating cell detachment from a primary site and disease dissemination [118,119]. The PI3K pathway and its downstream effectors have also been shown to induce the formation of lamellipodia and filopodia in response to HGF, thereby enhancing cell motility [120]. These cytoskeleton remodelers, as well as proteins involved in extracellular matrix (ECM) degradation (e.g., metalloproteases), have specifically been implicated in METex14-mediated cell invasion as well [121], providing valuable insight into METex14-specific signaling behavior. Other studies examining METex14 specifically have demonstrated its predilection for signaling through the MAPK pathway [122,123]; however, the specific mechanisms by which METex14 signals and mediates physiological processes within the cell require further exploration.

## 3. MET TKI Resistance

As mentioned earlier, various targeted therapies are either in development or have received clinical approval for the treatment of MET-driven NSCLC. The efficacy of these therapies in the clinic is often limited as patients present with either primary or acquired resistance. As a result, response rates for MET TKIs are relatively low compared to those for TKIs targeting other common NSCLC drivers [32,33,38,124,125,126].

### 3.1. On-Target Mutations

Both on- and off-target mechanisms of acquired resistance to MET TKIs have been observed. On-target mutations that mediate resistance to MET TKIs are able to reduce therapeutic efficacy by weakening the interaction between MET and the TKI. Different classes of TKIs are affected by mutations at different residues based on the nature of their interaction with the RTK (Table 2); for instance, resistance to type I TKIs can occur through mutations at D1228 and Y1230 (also designated as D1246 and Y1248 depending on the transcript on which the annotation is based [127]) and have been observed in vitro and in patients treated with both type IA and IB TKIs [102,128,129,130,131]. The type IA TKI crizotinib furthermore shows limited efficacy in instances where the G1163 residue is mutated [102,130]. Mutations at the aforementioned residues can still be treated with type II MET TKIs in an effort to overcome resistance; however, on-target mutations in other residues can still interfere with type II TKI effectiveness [131,132]; mutations affecting type II TKI activity have been observed in vitro at residues L1195 and F1200 [102].

### 3.2. Off-Target Mechanisms

Acquired resistance to MET TKIs occurs more often through the development of off-target mutations and bypass mechanisms than through on-target mutations [130,133]. Downstream pathway activation has commonly been found to occur through activating mutations in MAPK pathway effectors including KRAS and BRAF, while parallel pathway activation is often mediated through the amplification of ErbB family RTKs [130,133,134,135,136] (Table 3).

#### 3.2.1. RAS Pathway Activation

As a downstream effector of MET and a mediator of MAPK signaling, KRAS amplifications and mutations have both been implicated in bypass mechanisms that allow for resistance to MET TKIs. In the context of METex14-driven tumors, the prevalence of KRAS mutations as mechanisms of resistance to MET TKIs can be explained by the fact that METex14 preferentially activates the RAS-MAPK pathway to promote oncogenesis [122]. In tumor samples derived from patients harboring METex14 mutations, KRAS amplification occurred following treatment with crizotinib, and further investigation demonstrated that KRAS amplification rendered cells hypersensitive to growth factors and activation by RTKs other than MET; this allowed for the sustained signaling of MAPK and PI3K pathways during METex14 inhibition [137]. KRAS activity can furthermore be decoupled from METex14 signaling through mutations to KRAS itself. Substitution mutations at the twelfth codon position of the protein—commonly G12C, G12D, or G12V—result in the constitutive activation of KRAS, and consequent constitutive signaling through downstream MAPK and PI3K pathways. Combination therapies with MET TKIs and trametinib—an inhibitor of the MAPK pathway via the inhibition of MEK 1/2 activity—have shown a greater reduction of tumor growth in vitro than either therapy alone and suggest the utility of such therapeutic strategies for patients with concurrent MET and KRAS mutations [135,136]; however, tailoring this treatment approach for clinical use will require further investigation to determine optimal dosage for the patient to exhibit a response and lessen the severity of side effects [136].

#### 3.2.2. EGFR Signaling

Epidermal growth factor receptor (EGFR) is a member of the ErbB family of RTKs, and like MET, has been implicated in NSCLC development and activation of pathways that mediate cell proliferation and survival [144,145]. Some of the pathways whose activity is mediated by EGFR are likewise activated by MET, such as the MAPK and PI3K pathways; as such, aberrant activity of one receptor has been observed to compensate for the loss of the other. Amplification of EGFR is one of the common mechanisms of acquired resistance following MET TKI therapy [130,133], and METamp has likewise been implicated as a mediator of TKI resistance in EGFR-driven NSCLC [146,147,148]. In METamp NSCLC lines modeling acquired resistance to MET TKI, resistant clones displayed heightened dependency on EGFR, showing sensitivity to either EGFR inhibition alone or in combination with MET inhibition [138]. The opposite has likewise been observed, wherein EGFR TKI-resistant EGFR mutant cell lines were generated and found to rely upon METamp as a mechanism of resistance; MET-mediated maintenance of PI3K signaling via ErbB3 has been found to be a factor contributing to resistance in this instance [149]. Combinatorial therapeutic approaches targeting both EGFR and MET have been tested clinically—predominantly in the context of EGFR-driven cancers with MET-mediated acquired resistance—and have shown promising results, supporting the benefit of this approach for NSCLC treatment. Compared to standard chemotherapy, the combination of tepotinib and gefitinib was observed to improve both PFS and overall survival (OS) in patients who presented with EGFR mutant NSCLC and either high MET overexpression or amplification [150]. Furthermore, gefitinib has been evaluated in combination with capmatinib, which achieved an ORR of 47% in patients with a MET copy number greater than 6 and an ORR of 32% in patients with a MET overexpression status of IHC 3+ [151]. The combination of osimertinib and savolitinib in patients with EGFR-mutated and MET-amplified NSCLC was additionally evaluated in the TATTON trial. This approach achieved an ORR of 33% (95% CI, 22 to 46) in patients having been previously treated with a third-generation EGFR TKI and as high as 67% (95% CI, 41 to 87) in patients who had not previously been treated with a third-generation EGFR TKI [152]. Interestingly, while MET overexpression is often used to help predict therapeutic efficacy, studies have shown that receptor overexpression is not always a reliable measure of receptor activation, suggesting a need for better detection of receptor phosphorylation rather than protein level alone [153,154,155].

The dual targeting of EGFR and MET can furthermore be achieved through the administration of bispecific antibodies, such as amivantamab [156]. Amivantamab can disrupt the activity of both receptors by interrupting ligand binding, promoting receptor degradation, and directing immune cell activity [157,158,159,160]. In clinical trials, this antibody—when administered alone or in combination with other lines of treatment—has demonstrated improved PFS and ORR in patients with EGFR mutations. In the PAPILLON trial, patients with EGFR exon 20 insertions receiving amivantamab in combination with chemotherapy demonstrated improved median PFS compared to those receiving chemotherapy alone (11.4 months vs. 6.7 months, respectively) [161]. Amivantamab was also tested in combination with lazertinib in patients harboring EGFR exon 19 deletions or L858R mutations in the MARIPOSA trial; compared to patients who received osimertinib alone, the combination treatment achieved a similar ORR, but PFS was significantly longer (median of 23.3 months vs. 16.6 months) [162]. Promising results have also been observed with amivantamab being administered alone, as was observed in the CHRYSALIS trial, wherein amivantamab achieved an ORR of 40% (95% CI, 29 to 51) and median PFS of 11.1 months in patients with EGFR exon 20 insertions who previously received platinum chemotherapy [163]. Given the results of these trials, amivantamab has received approval for the treatment of NSCLC with EGFR exon 20 insertions, exon 19 deletions, and L858R mutations.

#### 3.2.3. PI3K Pathway Activation

Though mutations directly within the PI3K pathway have not been observed in the clinic as mechanisms of acquired resistance to MET TKIs, these mutations have been observed in cases of NSCLC and, specifically, cases of METex14-positive NSCLC [139,164]; as such, their potential role in mediating treatment resistance has been evaluated. Common “hotspot” mutations in PI3K, specifically in the p110α catalytic subunit (PIK3CA), have been observed to decouple this pathway from upstream RTK activation and promote transformation by enhancing PI3K activity [165]. Two such mutants—PIK3CA E545K and H1047R—have demonstrated an ability to promote MET TKI resistance in vitro as indicated by their ability to maintain downstream signaling following MET inhibition, and H1047R has also been shown to contribute to resistance in xenograft models [139,140]. The effect of either mutant on reducing sensitivity to MET inhibition, whether in vitro or in vivo, could be attenuated through the inhibition of both MET signaling and PI3K activity [139,140].

Mutations affecting PI3K pathway signaling may not always occur within effectors of the pathway but rather in pathway regulators. Phosphatase and tensin homolog (PTEN) is a regulator of the PI3K pathway, which works by antagonizing the effects of PI3K; while PI3K mediates the conversion of phosphatidylinositol 4,5-bisphosphate (PIP2) to phosphatidylinositol (3,4,5)-trisphosphate (PIP3) to further the signaling cascade, PTEN facilitates the reverse reaction [166,167]. In this capacity, PTEN functions as a tumor suppressor, and as such, loss of function mutations within this protein can enable constitutive signaling through the PI3K pathway. This was observed in vitro in cells displaying MET exon 14 skipping and PTEN loss, as treatment with a variety of MET TKIs was unable to inhibit signaling and cell survival in this line [139]. As was the case with PIK3CA mutant lines, the greatest reduction in signaling and survival was observed following dual inhibition of MET and PI3K [139]. The regulation of PI3K activity in combination with MET inhibition could, therefore, offer a potential mechanism of improving response to MET TKIs in MET-positive patients harboring PI3K pathway mutations; however, despite the promising observations in vitro and in vivo, further investigation is required to determine any benefit in the clinic.

#### 3.2.4. MYC Activation

The activation of signaling pathways by RTKs enables the activation of signaling cascades that ultimately result in the transcriptional activation of genes that regulate a variety of processes. One such regulator is MYC, a transcription factor that induces the expression of target genes necessary for various processes, including cell survival and proliferation; deregulation of MYC activity results in the dysregulation of downstream processes and contributes to oncogenesis in various tissues [168,169]. Sustained MYC activity can occur directly through signaling by the MAPK pathway, as MAPK is able to directly phosphorylate MYC at S62, resulting in the increased stability of this transcription factor [170]. In a similar fashion, the PI3K pathway promotes MYC activity by maintaining its stability; namely, PI3K recruitment to MET leads to the activation of AKT and the subsequent inhibition of GSK3β. This prevents GSK3β-mediated phosphorylation at T58, which normally targets MYC towards degradation [170,171].

Many of the aforementioned mechanisms of resistance to MET TKIs are, therefore, analogous in their ability to promote MYC activity. In concordance with this idea, studies of MET TKI resistance in NSCLC have demonstrated that while the specific mechanisms of resistance vary, they often result in dependence on MYC activity, both in vitro and in vivo [141,142]. In other malignancies, MYC and MET have been shown to cooperate to drive tumorigenesis, and their co-overexpression tends to exacerbate tumor growth and patient prognosis [143,172]. MYC silencing on the other hand has the opposite effect, slowing tumor progression, and when performed in combination with MET inhibition, it resensitizes resistant models to TKIs [141,142,143]. Targeting MYC directly in the clinic is challenging due to difficulties in effective drug design [173]; however, targeting bypass signaling pathways that regulate MYC as a convergent point of resistance may be an alternate approach to prevent its reactivation in MET-driven tumors. This underscores the need for a systems-level approach to delineate the regulatory relationship between MYC and upstream MET signaling.

## 4. Conclusions

While much headway has been made in expanding our understanding of METamp- and METex14-positive lung cancer, there are still shortcomings in our ability to target and treat patients with these malignancies. Though targeted therapy against MET is possible, resistance to these therapies results in their efficacy being much lower than what is observed for TKIs targeting other RTKs that drive lung cancer progression [32,33,38,124,125,126]. Furthermore, the prevalence of TRAES has previously resulted in patients requiring dose reductions or treatment discontinuation, further limiting treatment efficacy [174]. This results in a need for improved MET TKI design or the implementation of combination-based approaches to improve patient prognosis.

Designing and validating such therapies could be facilitated through the use of models displaying these mutations; however, while there are various cell lines available to this end, there is a current lack of animal models, particularly those displaying METex14-positive lung cancer. Current studies predominantly rely upon patient-derived xenografts (PDXs) or humanized mouse models that express HGF to study MET-positive cancers and MET-targeting therapeutics [154,175,176,177]. While such models are able to facilitate studies examining MET-driven oncogenesis and overcome challenges such as the lack of reactivity between murine ligand and human MET [178,179], they are limited in that they are unable to effectively recapitulate the tumor microenvironment of the malignancies being studied. To this end, transgenic mouse models for MET would be largely beneficial; such models have already been developed for other oncogenic drivers, and they have effectively modeled tumor biology and allowed for an improved investigation into mechanisms of therapeutic resistance [180,181,182]. Such models for MET could, therefore, further enable studies in therapeutic design and efficacy and help to better understand MET-driven tumor biology.

## Figures and Tables

**Table 1:** MET Tyrosine Kinase Inhibitors (TKIs).

TKI	Class	Specificity	Clinical Trials
Crizotinib	Ia	MET, ALK, ROS1	NCT00585195 [31,32]
Capmatinib	Ib	MET	NCT02414139 [33,34] NCT02750215 [35]
Savolitinib	Ib	MET	NCT02897479 [36,37]
Tepotinib	Ib	MET	NCT02864992 [38] NCT03940703 [39]
Glesatinib	II	MET, VEGFR1/2/3, RON, TIE-2	NCT02544633 [40]
Merestinib	II	MET, AXL, RON, MKNK1/2	NCT02920996 [41]
Cabozantinib	II	MET, RET, AXL, VEGFR1/2/3, FLT3, KIT	NCT01908426 [42]
Foretinib	II	MET, RON, AXL, TIE-2, VEGFR2, KIT, FLT3, PDGFRβ	NCT00742131 [43]
Tivantinib	III	MET, NLRP3	NCT01244191 [44] NCT01755767 [45]

**Table 2:** On-target MET mutations affecting the efficacy of MET TKIs.

Mutated MET Residue	TKI Class Affected	Citation
D1228/D1246 ^1^	Type I	[102,128,129,130,131]
Y1230/Y1248 ^1^	Type I	[102,128,129,130]
G1163	Type Ia	[102,130]
L1195	Type II	[102,130]
F1200	Type II	[102]

^1^ Transcript dependent [127].

**Table 3:** Off-target mechanisms of resistance to MET TKIs.

Alteration Conferring Resistance	Effect of Alteration	Mechanism of Resistance	Citations
KRAS amplification and mutation (e.g., G12C/G12D/G12V)	Constitutive KRAS signaling	Sustained MAPK and PI3K pathway activation, leading to increased survival and proliferation	[135,136,137]
EGFR-activating mutations	Constitutive EGFR signaling		[130,133,138]
PIK3CA mutations	Constitutive PI3K signaling	Sustained PI3K pathway activation and increased survival and proliferation	[139,140]
PTEN loss	Absence of PI3K signaling regulation	Increased PI3K pathway activity and signaling due to loss of negative regulation of PI3K	[139]
Aberrant MAPK and PI3K signaling	MYC stabilization	MYC overactivation and subsequent increased expression of MYC target genes	[141,142,143]

## Data Availability

The original data presented in this study are openly available in MSK-CHORD at https://doi.org/10.1038/s41586-024-08167-5.
